# Comparative Analysis of Testicular Histology and lncRNA–mRNA Expression Patterns Between Landes Geese (*Anser anser*) and Sichuan White Geese (*Anser cygnoides*)

**DOI:** 10.3389/fgene.2021.627384

**Published:** 2021-03-02

**Authors:** Mingxia Ran, Huaxuan Huang, Bo Hu, Shenqiang Hu, Jiwei Hu, Liang Li, Hua He, Hehe Liu, Jiwen Wang

**Affiliations:** Farm Animal Genetic Resources Exploration and Innovation Key Laboratory of Sichuan Province, Sichuan Agricultural University, Chengdu, China

**Keywords:** goose, lncRNA, transcriptome, testis, histology

## Abstract

Landes geese and Sichuan White geese are two important genetic materials for commercial goose breeding. However, the differences in the male reproductive capacity between these two breeds and the potential molecular mechanisms and associated key genes have not been reported to date. The present study compared the testicular histology and mRNA–long non-coding RNA (lncRNA) expression patterns to reveal the differences in male reproductive performance between Sichuan White geese and Landes geese, as well as to explore the underlying molecular mechanisms. Histological results showed that the testicular organ index, semen volume, and long diameter of seminiferous tubules of Landes geese were significantly larger than those of Sichuan White geese. Analyses of mRNA-lncRNA expression profile showed that compared with Sichuan White geese, a total of 462 differentially expressed mRNAs (DEGs) (173 up-regulated and 289 down-regulated) and 329 differentially expressed lncRNAs (DE lncRNAs) (280 up-regulated, 49 down-regulated) were identified in Landes geese. Among these DEGs, there were 10 spermatogenesis-related and highly expressed (FPKM > 10) DEGs. Except for *SEPP1*, all of these DEGs were significantly up-regulated in the testes of Landes geese. Functional enrichment analysis indicated that the pathway related to metabolism progress and phosphoinositol signal is vitally responsible for differences in male reproductive performance between Landes geese and Sichuan White geese. These results show that compared with Sichuan White geese, the spermatogenesis in the testis of Landes geese was more active, which may be mainly related to the inositol phosphate signal. These data contribute to a better understanding of the mechanisms underlying different male reproductive performances between Landes geese and Sichuan White geese. This knowledge might eventually provide a theoretical basis for improving male reproductive performance in geese.

## Introduction

Geese are the economically most important species of poultry around the world. However, the low reproductive performance of geese significantly restricts their industrial scale (Ding et al., 2014). Until now, most studies on goose reproductive performance were focused on females, although the male goose is also critical for goose reproduction (Brillard, 2003). Furthermore, heritability of male reproduction–related phenotypes (e.g., semen quality) was reported to be significantly higher than those of females among domestic animals (Berry et al., 2014). These results indicate the importance of understanding the male reproductive performance in geese.

The testis is an important male endocrine and reproductive organ, and the seminiferous tubules within the testis are the main site of spermatogenesis. Spermatogenesis is a complicated physiological process of male reproduction, which includes mitotic proliferation of spermatogonia, primary spermatocytes that undergo meiosis into round sperm, and the round sperm morphology differentiating into sperm (Brillard, 2003; Slowinska et al., 2011; Akhlaghi et al., 2014; Haseeb et al., 2019). These major steps are also involved in avian spermatogenesis (Aire, 2014), but more details of avian spermatogenesis, especially the spermatogenesis of geese, and its related molecular mechanism still need to be further studied (Aire, 2014). The diameter and number of testis’ seminiferous tubules as well as the number of interstitial and Sertoli cells in seminiferous tubules can affect the normal spermatogenesis and sperm function, thus further altering male fertility (Sharpe et al., 2003; Ford et al., 2006; García-Tomás et al., 2010). Moreover, several hormones that are secreted by the testes are also crucial for the initiation and regulation of spermatogenesis (Thurston and Korn, 2000; White-Cooper et al., 2009). Therefore, differences in testicular tissue structure and related gene expression may be the main reason for the observed differences in reproductive performance between Landes geese (LG) and Sichuan White geese (SWG).

Over the past decade, advanced transcriptomic technologies have enabled the identification of thousands of genes that are expressed differentially during spermatogenesis. This is a sign that spermatogenesis is accompanied by the continuous activation and/or inhibition of thousands of genes and proteins. Furthermore, a number of long non-coding RNAs (lncRNAs) are also dynamically expressed during spermatogenesis (Wichman et al., 2017). LncRNAs are transcripts similar to mRNAs but without protein-encoding capacity. Many lncRNAs were considered to be key regulators of gene expression, and their expression is generally tissue-specific; the testis is one of the organs with the highest number of expressed lncRNAs (Wichman et al., 2017). Furthermore, lncRNAs have been proven to participate in the proliferation, differentiation, and self-renewal of germ cells, as well as the induction of spermatogonial stem cells (Liu et al., 2018). These results indicate that the analysis of mRNA and lncRNA expression patterns is necessary to understand spermatogenesis and its regulatory mechanism in geese.

Both LG and SWG are important genetic resources that originated from different ancestors, with different genetic backgrounds. LG are famous for their high capacity and susceptibility for fatty liver production (Chen et al., 2017). SWG are a native breed of Sichuan province in China (Kang et al., 2014). Compared with other geese species, the female SWG show better reproductive performance (Feng and Sun, 2010; Kang et al., 2014). However, the differences in male goose reproductive capacities between both breeds and the related mechanisms and key genes have not been reported to date. Such information is conducive to providing a reference for improving the reproductive performance of male geese. Therefore, the current study comprised testicular histology to identify the differences of reproduction performance. Then, the testicular mRNA-lncRNA expression pattern was further analyzed to lay a foundation for a better understanding of the molecular mechanism of different reproductive performance between LG and SWG.

## Materials and Methods

### Ethics Approval

Six male SWG (735 days old) and six male LG (779 days old) were obtained from the waterfowl breeding experimental farm of Sichuan Agricultural University, China. The handling procedure of all experimental animals was approved by the Sichuan Agricultural University Animal Welfare Committee under DKY-B20141401.

### Animals and Sample Collection

All of these geese used for this experiment originated from the same batch of pure SWG and Landes geese, were healthy, and were raised under the identical conditions. After measuring the external genitalia and live weight, 12 geese were slaughtered (mean ± *SD* body weights: LG 4.25 ± 0.54, SWG 4.40 ± 0.35) and randomly divided into two groups: hematoxylin-eosin (HE) staining group and mRNA-lncRNA sequencing group: half of LG (*n* = 3) and SWG (*n* = 3) were used to collect a proportion of their left testes, which was immediately frozen in liquid nitrogen and stored at −80°C until RNA extraction. The intact left testes of the other three LGs (*n* = 3) and SWGs (*n* = 3) were fixed with 4% formaldehyde solution (Biosharp Life Science, China) for histological comparison.

### Histomorphological Observation

The samples for histological comparison were dehydrated and embedded by TSJ-II automatic closed tissue dehydrator (Changzhou Zhongwei Electronic Instrument Co., Ltd, Changzhou, China) and BMJ-III embedding machine (Zhongwei electronic instrument factory in Changzhou suburb, Changzhou, China), respectively. A rotary microtome (Leica, Oskar-Barnack, Germany) was used to cut the specimens into slices of 4 μm thickness. Cross-sections were further stained with HE and photographed with a digital trinocular camera microscope BA410 Digital (Motic China Group Co. Ltd., Xiamen, China). The long and short diameters of single seminiferous tubules and the number of germ cells they contained were counted by Image-Pro Plus 6.0 (Media Cybernetics Company of America, MD, United States).

### Total RNA Extraction, Library Preparation, and Sequencing

The Trizol kit (Invitrogen, CA, United States) was used to extract the total RNA of testis according to the manufacturer’s instructions. RNA purity was assessed using the NanoPhotometer^®^ spectrophotometer (IMPLEN, CA, United States). The Qubit^®^ RNA Assay Kit in Qubit^®^ 2.0 Fluorometer (Life Technologies, CA, United States) was used to assess the RNA concentration. The RNA integrity was assessed by RNA Nano 6000 Assay Kit of the Bioanalyzer 2100 System (Agilent Technologies, CA, United States).

A total amount of 20 ng RNA per sample was used as input material for RNA sample preparations. Firstly, Epicenter Ribo-Zero^TM^ rRNA Removal Kit (Epicenter, WI, United States) was used to remove ribosomal RNA, and rRNA-free residue was cleaned up by ethanol precipitation. Subsequently, sequencing libraries were built using the rRNA-depleted RNA by NEBNext^®^ Ultra^TM^ Directional RNA Library Prep Kit for Illumina^®^ (New England Biolabs, Inc., MA, United States) under the following the manufacturer’s recommendations. Briefly, fragmentation was performed using divalent cations at elevated temperature in NEBNext First Strand Synthesis Reaction Buffer (5X). The first-strand cDNA was synthesized through random hexamer primer and M-MuLV Reverse Transcriptase (RNaseH-). Then, the second-strand cDNA synthesis was performed by DNA polymerase I and RNase H. After that, dNTPs with dTTP were replaced by dUTP in reaction buffer. Next, the remaining overhangs were converted into blunt ends via exonuclease/polymerase activities. Finally, after the adenylation of 3′ ends of DNA fragments, NEBNext adaptor with a hairpin loop structure was ligated to prepare for hybridization. To preferentially select 150–200 bp cDNA fragments, the AMPure XP system (Beckman Coulter, Beverly, MA, United States) was used to purify the library fragments. A total of 3 μL USER Enzyme (New England Biolabs, Inc., MA, United States) was used for size selection. Before the polymerase chain reaction (PCR) reaction, adaptor-ligated cDNA was incubated at 37°C for 15 min followed by 5 min at 95°C. Then, Phusion High-Fidelity DNA polymerase, Universal PCR primers, and Index (X) Primer were added for PCR. At last, after purifying (AMPure XP system) the products, the quality of the library was assessed on the Agilent Bioanalyzer 2100 system. Before sequencing, the index-coded samples were clustered on a cBot Cluster Generation System using TruSeq PE Cluster Kit v3-cBot-HS (Illumina). After cluster generation, paired-end reads were generated by the sequencing of libraries on an Illumina Hiseq 2500 platform. All obtained RNA and lncRNA-seq data in the current study are available from the BioProject database (PRJNA669971).

### Quality Analysis, Mapping, and Transcriptome Assembly

Raw data (raw reads) in fastq format were first processed through in-house perl scripts. In this step, clean data (clean reads) were obtained after removal of reads containing adapter, reads on containing ploy-N, and low-quality reads. At the same time, Q20, Q30, and GC content of the clean data were calculated. All the downstream analyses were based on clean data. The genome of Swan geese (*Anser cygnoides*) (available at https://www.ncbi.nlm.nih.gov/genome/31397) was used as reference genome for both sequence alignment and subsequent analysis. Then, clean reads were mapped to the Swan geese (*A. cygnoides*) genome through HISAT2 (v2.0.4) (Langmead and Salzberg, 2012). The mapped reads of each sample were assembled by StringTie (v1.3.3) (Pertea et al., 2016) using a reference-based approach.

### Identification of lncRNAs

Based on the results of transcriptome splicing, a series of strict screening conditions were applied for lncRNA identification. First, after a large number of single-exon transcripts with low expression and low reliability had been filtered, transcripts with more than two exons were selected. Next, transcripts with a length >200 bp were further selected. This was followed by removal of the transcripts that overlap with the exon region of the database annotation. The lncRNAs that overlapped with the exon region of the splicing transcript in the database were included in the subsequent analysis as lncRNA annotated in the database. Subsequently, the expression of each transcript was calculated by StringTie (v2.1.1), and the transcript was selected with fragments per kilobase of exon per million fragments mapped (FPKMs) ≥1. Finally, the coding potential of the spliced transcripts was analyzed using four software packages: CPC2, CNCI, Pfam, and phyloCSF. And then, the intersection of non-coding transcripts was taken as identified by these four software packages.

### Differential Expression Analysis

Ballgown was used to calculate the FPKMs of both lncRNAs and coding genes in each sample. For biological replicates, *P* < 0.05 and an absolute log2 value (fold change) >1 were considered to indicate significant differences in mRNA or lncRNA expressions.

### Target Gene Prediction of lncRNAs

Protein-coding genes located at ∼10 kb upstream and downstream of lncRNA were classified as *cis*-target genes of lncRNA. Then, if the mRNA had a Pearson correlation coefficient with lncRNA that exceeded 0.95 or that was lower than −0.95 and had a *P* < 0.05, this mRNA was identified as a *trans*-target gene.

### Quantitative Real-Time PCR Validation

Four differentially expressed mRNA (DEGs) and five differentially expressed lncRNAs (DE lncRNAs) related to spermatogenesis that target these five mRNAs were selected for PCR verification. The total RNA from the testis of three LGs and three SWGs was reverse transcribed into cDNA using the PrimeScript^TM^ RT Reagent Kit (Takara Biotechnology Co., Ltd., Dalian, China). Furthermore, the quantitative PCR (qPCR) reactions were performed on the Bio-Rad CFX96 real-time PCR detection system (Bio-Rad, Hercules, CA, United States) using TB Green^TM^ Premix Ex Taq^TM^ (Takara Biotechnology Co., Ltd., Dalian, China) and the primer designed by Primer3Plus^1^ (Table 1). Relative expression levels were calculated using the comparative Cq method (ΔΔCq) method, and the statistical differences of genes were analyzed using one-way analysis of variance test in the SPSS (version 20.0, IBM, IL, United States). All data were shown as the means ± SEM. *P* < 0.05 was regarded to indicate statistically significant differences.

**TABLE 1:** Quantitative real-time PCR primers.

Gene name	Forward primer (5′–3′)	Reversed primer (5′–3′)	Product length (bp)
CAPZA3	TGACTCGGGTTACAGGGGTAT	ACATGCAGAGACGCACTTACA	100
LRRC18	CACAGGCTGTGGACAGAGAAT	GAAAATGGCAGCCCTTCAAGG	116
SEPP1	AATTCTTGCAGTTGACACGGC	CACTGTCAGGTGTCGCTAGTT	128
TSSK6	TCCAGCAAATACAAGGTCCCC	GATGAACTCGAAGATGCGCAC	147
LOC106036070	GGACTTCTCCTCTTGCCTCTG	AATGTTCAAACCGTGCTCTGC	119
LOC106035363	GTTAGGTGCAGAAAAGTGCCG	CTGTGTCCCAGGGAACTCTTC	112
XLOC_058157	AAGCTGAAAGATCGTTGTTGGC	TGCACCGAAGACATAACAGTGA	140
LOC106038337	TTCCAGGTGCCTCGTCAAAA	ACCAGCAAAGTGAGCCATCA	105
XLOC_169473	CCTTCTCACCTCGGCATGTT	AGCCTAGAGGGATCCGGTTT	94
GAPDH	TTTCCCCACAGCCTTAGCA	GCCATCACAGCCACACAGA	90
β-actin	CAACGAGCGGTTCAGGTGT	TGGAGTTGAAGGTGGTCTCG	92

### Gene Ontology and Kyoto Encyclopedia of Genes and Genomes Enrichment Analyses

Gene Ontology (GO) and Kyoto Encyclopedia of Genes and Genomes (KEGG) enrichment analyses of both DEGs or DE lncRNAs target genes were tested by KOBAS 3.0 online^2^ (Mao et al., 2005). GO terms and KEGG pathways with *P* < 0.05 were considered significantly enriched by the target genes of DEGs or DE lncRNAs.

## Results

### Comparative Analysis of the Testicular Histology Between Sichuan White Geese and Landes Geese

**FIGURE 1:**
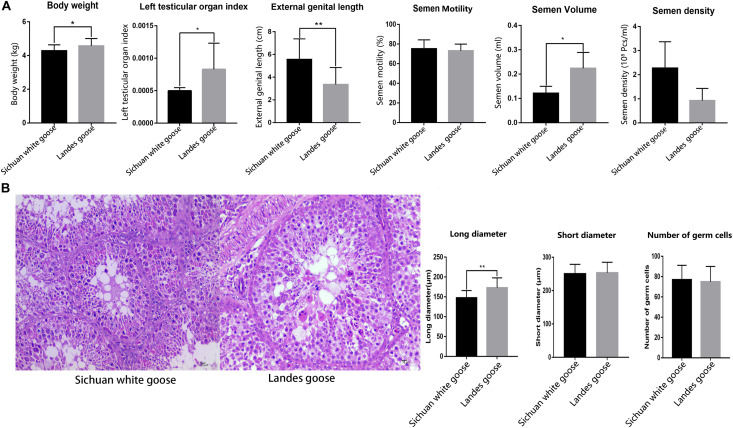
The histological observation and the counting of the long and short diameters of seminiferous tubules and the number of germ cells located in single seminiferous tubules. **(A)** Comparing the body weight (*n* = 6), length of the external genitalia (*n* = 6), testicular organ index (*n* = 6), and semen quality (*n* = 4) of Lande goose and Sichuan white goose. **(B)** HE staining of the testis (*n* = 3); black arrow points to Sertoli cells, and the green arrow points to germ cells (left), and measurement of the long and short diameters of seminiferous tubules and number of germ cells located in single seminiferous tubules (right). * means significant difference, ** means extremely significant difference.

Comparing the body weight, length of external genitalia, testicular organ index, and semen quality of LG and SWG showed that the testicular organ index (*P* = 0.03) and semen volume (*P* = 0.03) of LG were higher than that of SWG. However, the length of external genitalia was significantly shorter than that of SWG (*P* = 0.00017) (Figure 1A). Histological results showed that the long diameter of seminiferous tubules of LGs significantly exceeded that of SWGs (*P* = 0.000194) (Figure 1B). These results corroborated the difference in the reproductive performance of male goose between male LG and SWG.

### Overview of Sequencing Data and lncRNA Identification

To explore the global lncRNA–mRNA expression profiles, the total RNAs of the testes of three LGs and three SWGs were extracted for lncRNA–mRNA sequencing. Integrity and purity of RNA were detected by agarose gel electrophoresis and Agilent 2100, respectively. The results indicate that the purity and integrity of RNA extracted from these samples were sufficiently good to be used for subsequent library construction and sequencing (Supplementary Figure 1). Applying to a strict filtering condition, LG (*n* = 3) and SWG (*n* = 3) yielded an average of 100,190,736 and 96,778,761 clean reads, respectively, and the Q30 of each sample was no less than 93% (Supplementary Table 1). The Swan goose genome was used as the reference genome; the average mapping rates of LG and SWG were 73.84 and 75.69%, respectively.

A stringent filtering pipeline (size > 200 bp, number of exons ≥ 2) was adopted to identify reliable candidate lncRNAs from assembled transcripts. As a result, a total of 33,484 protein-coding genes and 4,458 annotated lncRNAs [most lncRNAs were sense intergenic lncRNAs (50.2%), followed by intronic-lncRNA (36.2%) and antisense lncRNAs (13.6%)] (Supplementary Figure 2), as well as 27,938 novel lncRNAs, were identified. These lncRNAs had similar expression characteristics with other research (Supplementary Figure 3; Knoll et al., 2015).

### Differentially Expressed mRNAs and lncRNAs in the Testes Between Sichuan White Geese and Landes Geese

**FIGURE 2:**
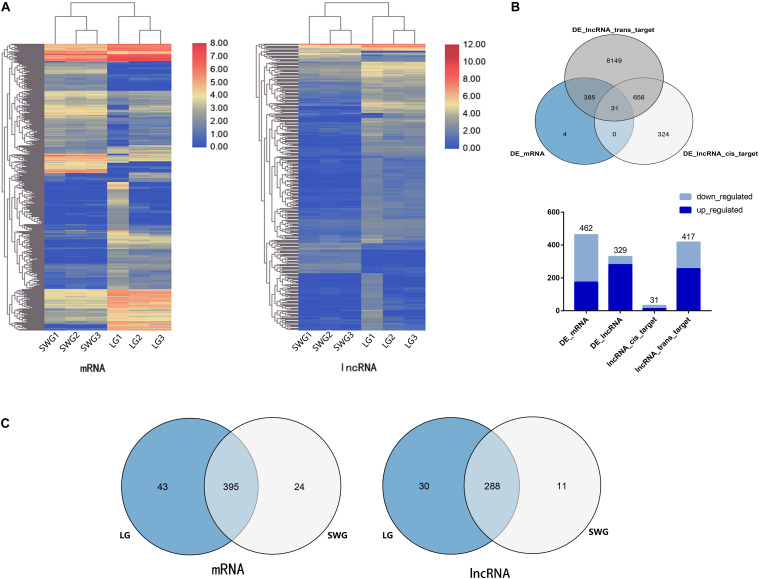
Analysis of differentially expressed mRNA and lncRNA. **(A)** Cluster map of differentially expressed mRNA and lncRNA. **(B)** Venn diagram of DEGs and the target of DE lncRNAs, followed by the histogram of the up-regulation and down-regulation distribution of DEGs, DE lncRNAs, and the target DEGs of DE lncRNAs. **(C)** Venn diagram of DEGs and DE lncRNAs in Landes (LG) and Sichuan White geese (SWG).

To investigate the difference in the expression patterns of lncRNA–mRNA in the testis between LG and SWG, DEGs, and DE lncRNAs were screened out under the conditions of log2 (fold change) >1 and *P* < 0.05. Compared with SWG, a total of 462 DEGs (173 up-regulated and 289 down-regulated) and 329 DE lncRNAs (280 up-regulated and 49 down-regulated) were obtained from the LG (Figure 2A). These 329 DE lncRNAs had a total predicted 1,018 *cis*-target genes and 9,221 *trans*-target genes (Supplementary Table 2). Among these target genes, 31 *cis*-target genes and 417 *trans*-target genes were differentially expressed between LG and SWG. A total of 31 DEGs were predicted to be both *cis-* and *trans*-target genes of DE lncRNAs (see Figure 2B). Furthermore, 43 DEGs and 30 DE lncRNAs were specifically expressed in LG; 24 DEGs and 11 DE lncRNAs were specifically expressed in SWG (Figure 2C).

**FIGURE 3:**
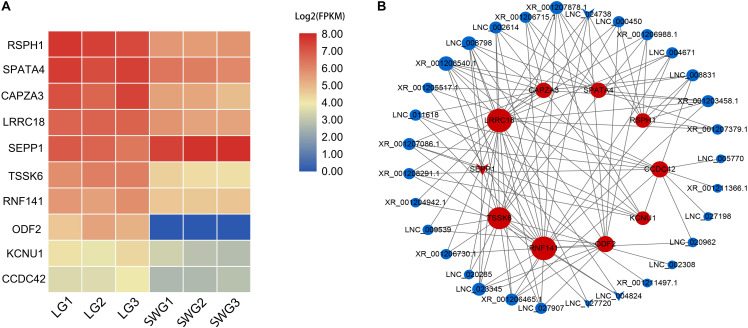
Heatmap of spermatogenesis-related DEGs **(A)** and their interaction networks with corresponding DE lncRNAs **(B)**. **(B)** Blue point represents the lncRNA; red point represents the target mRNA; the size of the dot indicates the number of lncRNA or mRNA that has a targeting relationship with the gene represented by this dot; the gray lines represent transregulatory relationships, and the “round” shape indicates that the mRNA or lncRNA is up-regulated in LG; “V” indicates that it is down-regulated in LG.

According to the functional annotation of the Genecard database, 19 DEGs were found to be related to spermatogenesis. Among these, the FPKM value of 10 DEGs was greater than 10 (Figure 3A). Except for SEPP1, the other nine DEGs were up-regulated in LG testis (Table 2). These 10 DEGs were targeted by 38 DE lncRNAs with FPKM > 10, and most of these DE lncRNAs were also up-regulated in LGs (Figure 3B and Supplementary Table 3).

**TABLE 2:** Differentially highly expressed mRNA related to spermatogenesis.

Gene name	log2 (fold change)	*P*-value	Function
RSPH1	1.89	0.02	May play an important role in male meiosis
SPATA4	1.27	0.04	Spermatogenesis-associated 4; may play a role in apoptosis regulation
CAPZA3	1.93	0.01	May play a role in the morphogenesis of spermatid
LRRC18	1.24	0.04	May be involved in the regulation of spermatogenesis and sperm maturation
SEPP1	–1.03	0.01	May supply selenium to the testis
TSSK6	1.86	0.02	Required for sperm production and function
RNF141	1.03	0.01	May be involved in spermatogenesis
ODF2	–	0.01	Seems to be a major component of sperm tail outer dense fibers
KCNU1	1.22	0.03	Testis-specific potassium channel
CCDC42	1.13	0.04	Required for sperm development

### Functional Analysis of DEGs and Predicted Target Genes of DE lncRNAs

**FIGURE 4:**
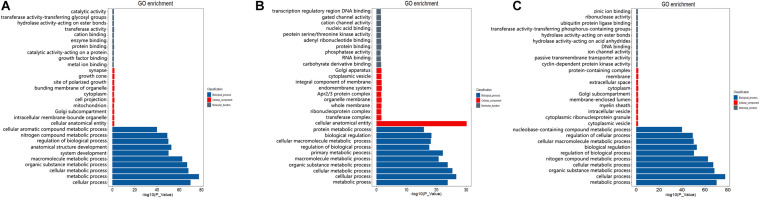
GO enrichment analysis of DEGs and target genes of DE lncRNAs. The top 10 GO terms with the most significant enrichment of DEGs **(A)**, *cis*-target **(B)**, and *trans*-target **(C)** in biological process, cellular components, and molecular function, respectively.

To preliminarily explore the potential regulatory mechanism of different spermatogenesis between LGs and SWGs, functional enrichment analysis was conducted. The result indicated that 462 DEGs were significantly enriched in 614 GO terms (*P* < 0.05) (Figure 4A). Totals of 1,018 *cis*-target genes and 9,221 *trans*-target genes of lncRNAs were significantly enriched in 1,118 and 1,678 GO terms, respectively (Figures 4B,C). Furthermore, both DEGs and the target genes of DE lncRNAs were mainly enriched in metabolism-related GO terms (metabolic process, organic substance metabolic process, cellular metabolic process, nitrogen compound metabolic process). This indicates that most DEGs and target genes of these DE lncRNAs were related to metabolic processes.

**FIGURE 5:**
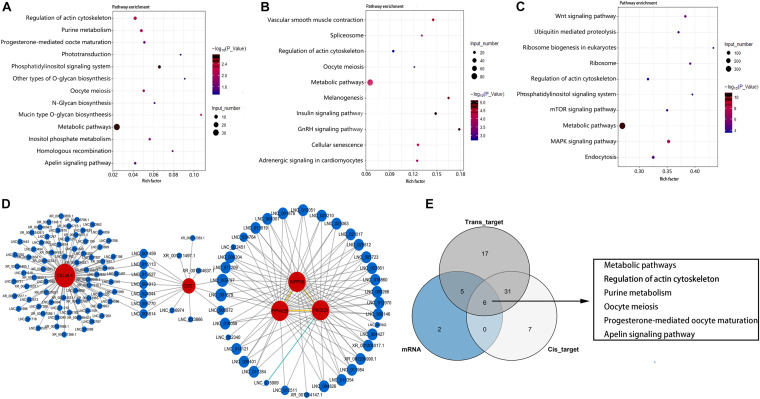
KEGG enrichment analysis. The 13 pathways significantly enriched by DEGs **(A)** and the top 10 pathways with the most significant enrichment of lncRNA *cis-*target **(B)** and *trans-*target **(C)** genes. Point color represents the significance of enrichment, and point size represents the number of differentially expressed genes enriched in each pathway. **(D)** The corresponding DE lncRNAs of the DEGs enriched in the phosphatidylinositol signaling system pathway. **(E)** The Venn diagram of the pathways enriched by DEGs and the target genes of DE LncRNAs.

KEGG enrichment analysis showed that these DEGs were significantly enriched in 13 pathways (*P* < 0.05) (Figure 5A). These include metabolism-related pathway (metabolic pathways, purine metabolism, mucin type O-glycan biosynthesis, N-glycan biosynthesis, other types of O-glycan biosynthesis), inositol phosphate (InsP) signal-related pathway (phosphatidylinositol signaling system, InsP metabolism, and regulation of actin cytoskeleton), and germ cell development–related pathways (oocyte meiosis, homologous recombination, and progesterone-mediated oocyte maturation). The top three most enriched pathways were phosphatidylinositol signaling system, metabolic pathways, and regulation of actin cytoskeleton pathway. As the most significantly enriched pathway, the phosphatidylinositol signaling system conducts critical signaling during spermatogenesis. Furthermore, all DEGs enriched in this pathway were up-regulated in LG (Supplementary Table 4). These results suggest that InsP signaling may play an important role for the differences in reproductive performance between LG and SWG. Therefore, an interaction network for DEGs enriched in the phosphatidylinositol signaling system pathway and its corresponding DE lncRNAs was built to provide a reference for the regulatory role of lncRNA. As shown in Figure 5D, 114 DE lncRNAs participated in regulating the DEGs (17 DE lncRNAs’ FPKM > 10), and all of which were up-regulated in LG. Furthermore, CALML4, which had the highest expression level among the genes enriched in the phosphoinositide signaling system pathway, was targeted by 80 DE lncRNAs (Figure 5D). Except for this, all of the DEGs enriched in oocyte meiosis and progesterone-mediated oocyte maturation were also up-regulated in LG (Supplementary Table 4). In addition, *cis*- and *trans*-target genes of DE lncRNAs were also significantly enriched in 44 and 59 KEGG pathways, respectively. The top three most enriched pathways by the *cis*-target genes of DE lncRNAs were metabolic pathways, mitogen-activated protein kinase (MAPK) signaling pathway, and insulin signaling pathway (Figure 5B). Those among the *trans*-target gene–enriched pathway of DE lncRNAs were metabolic pathways, MAPK signaling pathway, and neuroactive ligand–receptor interaction (Figure 5C). Consistent with the GO enrichment analysis, the pathway with the largest number of DEGs and target genes of DE lncRNAs also was the metabolic pathway. This suggests that the difference of spermatogenesis between LG and SWG mainly manifests in metabolic processes.

Combined analysis of DEGs and target genes of DE lncRNA-enriched pathways showed that six pathways were simultaneously enriched by DEGs and both the *cis*- and *trans*-target genes of DE lncRNAs: metabolic pathways, regulation of actin cytoskeleton, purine metabolism, oocyte meiosis, progesterone-mediated oocyte maturation, and apelin signaling pathway (Figure 5E). This indicates that these pathways may be critical for lncRNA regulating the difference of spermatogenesis between LG and SWG. It is worth mentioning that PIK3CB, which is enriched in the phosphatidylinositol signaling pathway, is also simultaneously enriched in the InsP metabolism pathway, metabolic pathways, and progesterone-mediated oocyte maturation. Furthermore, it was expressed specifically in the testes of LG. These results suggest that PI3KCB may play a critical role in the interaction of these pathways involved in regulating the differences between spermatogenesis of LG and SWG.

### Validation of DEGs and DE lncRNAs

**FIGURE 6:**
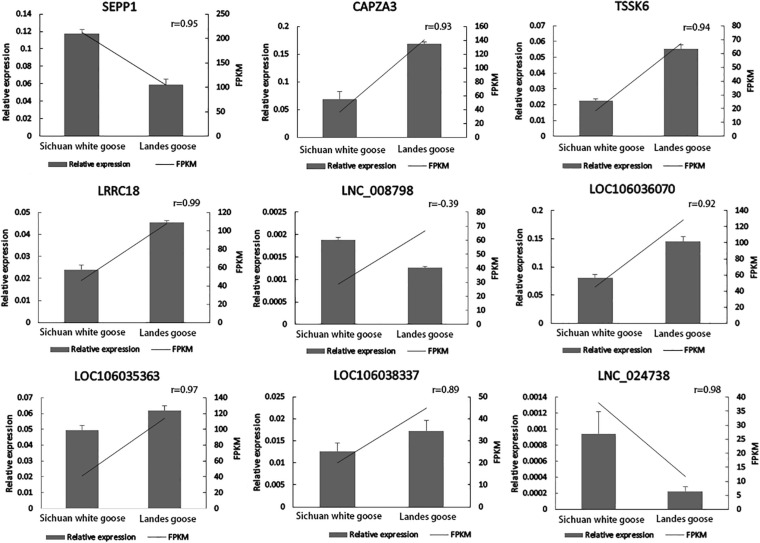
Verification of expression patterns of four DEGs and five DE lncRNAs.

Four DEGs involved in spermatogenesis (*SEPP1*, *CAPZA3*, *TSSK6*, and *LRRC18*) and five of their corresponding DE lncRNAs (*XLOC_058157*, *LOC106036070*, *LOC106035363*, *LOC106038337*, and *XLOC_169473*) were selected for qPCR to verify expression profiles of these genes in the testes of LG and SWG. As shown in Figure 6, except for *XLOC_058157*, the other eight genes had expression patterns similar to the sequencing results. Consistent expression patterns of these eight genes corroborated the reliability of the RNA-Seq data.

## Discussion

Spermatogenesis, which is a highly sophisticated process that is part of the transmission of genetic heritage, is critical for the reproductive performance of male animals (Slowinska et al., 2011; Chocu et al., 2012). As an important reproductive and endocrine organ in males, the testis is critical for spermatogenesis (Ding et al., 2016). Studies on male boars have demonstrated that testicular histological parameters have stronger heritability than semen characteristics (Chang et al., 2017); therefore, domestic animals that differ in male reproductive capacity usually also show differences in their testicular structures. In pigs, Ding et al. found that the testis volume in Meishan boars with higher litter size was smaller than that in Duroc boars; moreover, the diameters of seminiferous tubules were significantly larger than those of Duroc boars at day 75 (Ding et al., 2016). The results of the present study showed that the testicular organ index and the long diameter of seminiferous tubules of LG significantly exceeded that of SWG. Research in boars has demonstrated that the testis size and the diameter of seminiferous tubules are positively correlated with sperm production (Walker et al., 2004). This suggests that the differences in testicular structures between LG and SWG may further cause differences in spermatogenesis.

The process of avian spermatogenesis, especially the spermatogenesis of geese and its related molecular mechanism, still needs to be further studied. In the present study, key genes in the spermatogenesis of geese were identified through transcriptome sequencing of LG and SWG testes. Ten DEGs that were highly expressed in goose testis were related to spermatogenesis. Among them, Tcte3 [radial spoke head component 1 (RSPH1)], which had the highest expression, is an accessory component of axonemal and cytoplasmic dynein, which expresses predominantly in meiotic and postmeiotic germ cells (Parveen et al., 2014). The others DEGs, *SPATA4*, and *RNF141*, play a role in the maintenance of spermatogenesis (Zhang et al., 2001; Jiang et al., 2013), whereas *SEPP1* and *LRRC18* (*mtLR1*) participate in the regulation of spermatogenesis and in sperm maturation (Nie et al., 2005; Michaelis et al., 2014; Jha et al., 2017); furthermore, *CAPZA3*, *TSSK6*, *ODF2*, *KCNU1*, and *CCDC42* were reported to be involved in regulating sperm morphogenesis (Sosnik et al., 2010; Leonetti et al., 2012; Pasek et al., 2016; Zhao et al., 2018; Czubaszek et al., 2019). All these spermatogenesis-related genes, except for *SEPP1*, were up-regulated in LG. This further suggests that LG showed higher spermatogenesis activity, especially sperm morphogenesis. Furthermore, research has shown that sex hormone production, spermatogenesis, and histological morphology of the testis change with age (Gunes et al., 2016). *TSSK6* was found to be required for γ*H2AX* formation during spermiogenesis and participated in the histone-to-protamine transition through γ*H2AX*. This transition of histone to protamine is the most important epigenetic process that takes place during spermiogenesis. This process is involved in the packing of the sperm genome into the sperm head, and active throughout spermatogenesis. With increasing age, the replacement of histone by protamine is increasingly obstructed, and abnormal retention of histone leads to an increase of DNA fragment, which further obstructs the spermatogenesis (Czubaszek et al., 2019). Therefore, the different expression of TSSK2 suggested that the difference in spermatogenesis between LG and SWG may be age related.

KEGG enrichment analysis showed that DEGs between LG and SWG were most significantly enriched in the phosphatidylinositol signaling system pathway in the testis. Furthermore, compared with SWG, all genes enriched in this pathway were significantly up-regulated in LG testes. These results indicate that the difference in reproductive performance between LG and SWG may be related to the difference of phosphoinositide signal activity in the testis. Phosphatidylinositol signaling was involved in nearly all important cellular processes including metabolism, membrane trafficking, cell proliferation, differentiation, apoptosis, and many others (Poli et al., 2016). Phosphatidylinositol phosphorylates the 3, 4, 5 position of the inositol ring to produce seven different phosphoinositide messengers [InsP or phosphatidylinositol phosphates (PIPs)] (Gross, 2017). The InsP metabolism pathway is also significantly enriched by DEGs. *PIP* levels are an important regulation signaling for germline cell maintenance, proliferation, and survival and also regulate spermatocyte cytokinesis, spermatid polarization, sperm tail formation, nuclear shape, and production of mature, motile sperm (Brill et al., 2016). These reports suggest that, similar to mammals, InsP also plays an important role in the regulation of spermatogenesis in geese. Furthermore, calmodulin-like 4 (CALML4), the gene with the highest expression level among the DEGs that are enriched in the phosphatidylinositol signaling pathway, may play an important role in the regulation of phosphatidylinositol-signaling-mediated spermatogenesis. CALML4 was reported to be a calmodulin-like protein and encoded a 153-amino-acid isoform, which share ∼45% identity with the 149-amino-acid sequence of calmodulin; similar to calmodulin, CALML4 also largely consists of four EF-hand motifs (Kapustina and Cheney, 2020). This further supports the hypothesis that the function of CALML4 may be consistent with that of calmodulin. In the phosphatidylinositol signaling system pathway, CALML4 promotes the phosphorylation of inositol (1,4,5) trisphosphate (IP3) into inositol (1,3,4,5) tetrakisphosphate [Ins(1,3,4,5) P4 (IP4)] via inositol triphosphate 3-kinase. InsPs are a highly diverse group of molecules, at least 30 of which occur in mammalian cells. Following its release from the lipid phosphatidylinositol 4,5-bisphosphate, IP3 can be further metabolized to more highly phosphorylated InsPs, which have diverse function in chemotaxis, vesicular trafficking, channel regulation, cell proliferation, and a variety of nuclear functions (González et al., 2004). In rat sperm, it is suggested that the flow of sperm calcium ions is directly mediated by InsP3 (Harrison et al., 1990). Therefore, CALML4 may be a key gene mediating the phosphatidylinositol signaling pathway and regulating the differences in spermatogenesis between LG and SWG.

In addition to these two InsP signal-related pathways mentioned above, DEGs were also significantly enriched in the regulation of the actin cytoskeleton pathway. This pathway was also significantly enriched by both the *cis*- and *trans*-target genes of DE lncRNA. Actin is distributed in the head, equator, posterior parietal region, and tail of sperm, indicating that actin plays an important role in the process of capacitation, acrosome exocytosis, and sperm movement. The increasing actin filaments in the head and flagellum during capacitation are crucial for hyperactivation (Breitbart and Finkelstein, 2018). It has been reported that phosphatidylinositol 4,5-diphosphate [PI (4,5) P2] can regulate the activity of many actin-binding proteins, including actin, cofilin, dia2, N-WASP, ezrin, and moesin. Sperm total motility and hyperactivated motility are mediated by PLD-dependent actin polymerization. The reduction of *PIP2* synthesis inhibits actin polymerization and sperm motility, whereas the increase of *PIP2* synthesis enhanced this activity (Senju et al., 2017). This further highlights the important role of phosphoinositide signaling in the regulation of spermatogenesis in geese through the regulation of the actin cytoskeleton pathway. Among the DEGs that are enriched in the regulation of the actin cytoskeleton pathway, *PIK3CB* is also enriched in the phosphatidylinositol signaling system pathway, suggesting that its potential involvement in the phosphatidylinositol regulating the actin cytoskeleton. Phosphatidylinositol-4,5-bisphosphate 3-kinase catalytic subunit β, a class of phosphoinositide-3-kinase (*PI3K*), phosphorylates PtdIns (phosphatidylinositol), PtdIns4P (phosphatidylinositol 4-phosphate), and PtdIns(4,5)P2 (phosphatidylinositol 4,5-bisphosphate) to phosphatidylinositol 3,4,5-trisphosphate (PIP3) (Jia et al., 2008). PIP3 plays a key role in activating signaling cascades involved in cell growth, survival, proliferation, motility, and morphology as it recruits PH domain-containing proteins to the membrane, including *AKT1* and *PDPK1*. PIP3 signaling is also required in spermatogonia for the initiation of meiotic progression and germ cell survival (Tates, 1971; Jia et al., 2008). As mentioned previously, 5 of the 10 DEGs related to spermatogenesis are related to sperm morphogenesis; the phosphatidylinositol signal may affect morphogenesis during spermatogenesis by regulating the actin cytoskeleton through *PIK3CB*.

## Data Availability Statement

The data presented in the study are deposited in the BioProject database, accession number is PRJNA669971.

## Ethics Statement

The animal study was reviewed and approved by the Sichuan Agricultural University Animal Welfare Committee. Written informed consent was obtained from the owners for the participation of their animals in this study.

## Author Contributions

JW and SH designed the study. MR, HH, BH, and JH completed the majority of the experiments. BH weighted and executed the animals by cervical dislocation. JH was involved in the dissecting of the animals. HH weighed the testicles. MR was responsible for the collection of left testicles and participated in data analysis and wrote the manuscript. JW, SH, HL, and LL participated in manuscript revision and editing. All authors read and approved the final manuscript.

## Conflict of Interest

The authors declare that the research was conducted in the absence of any commercial or financial relationships that could be construed as a potential conflict of interest.

## Footnotes

1 http://primer3plus.com/

2 http://kobas.cbi.pku.edu.cn/kobas3/?t=1
