# Supplementary material for: Comparative Analysis of Testicular Histology and lncRNA–mRNA Expression Patterns Between Landes Geese (Anser anser) and Sichuan White Geese (Anser cygnoides)
Source: Front Genet. 2021 Mar 2;12:627384. doi: 10.3389/fgene.2021.627384 (PMC7963104; doi:10.3389/fgene.2021.627384)
Supplement: Supplementary file 1 [file Table_1.DOCX]

Supplementary Material

# Supplementary Figures and Tables


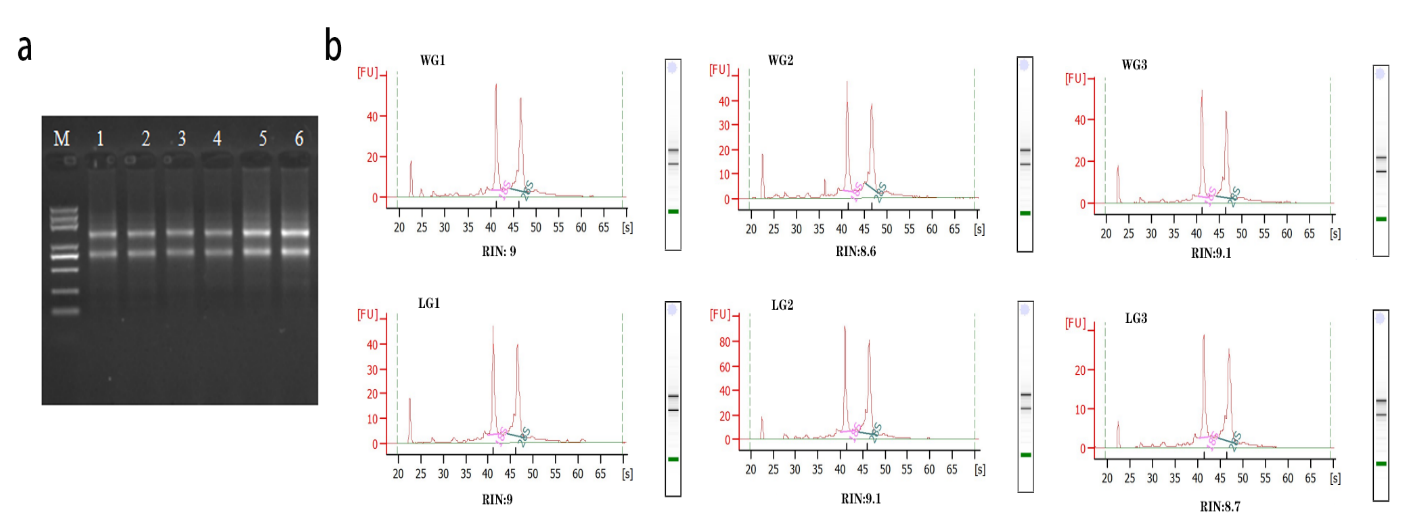


**Supplementary Figure 1.** RNA integrity and purity analyzes of testis. a: Agarose gel electrophoresis of RNA extracted from testis. b: Detection of RNA integrity by Agilent 2100 system.


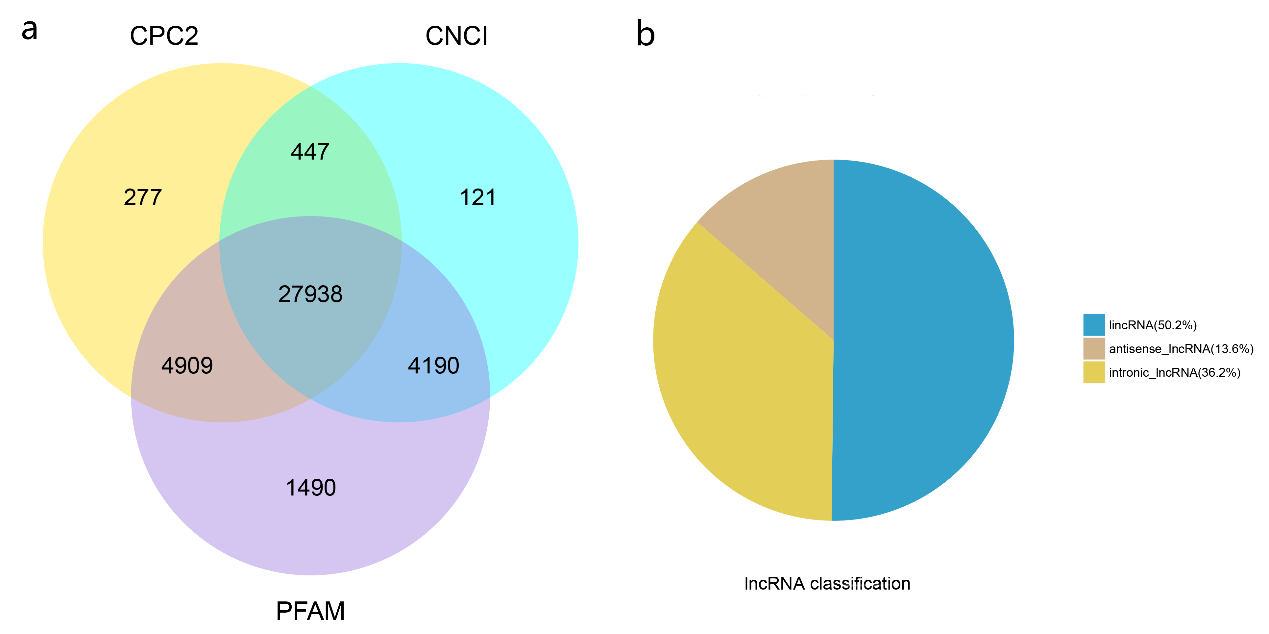
**Supplementary Figure 2.** Identification of lncRNA a: Wayne map of CPC2, CNCI, PFAM coding potential analysis; b: lncRNA classification


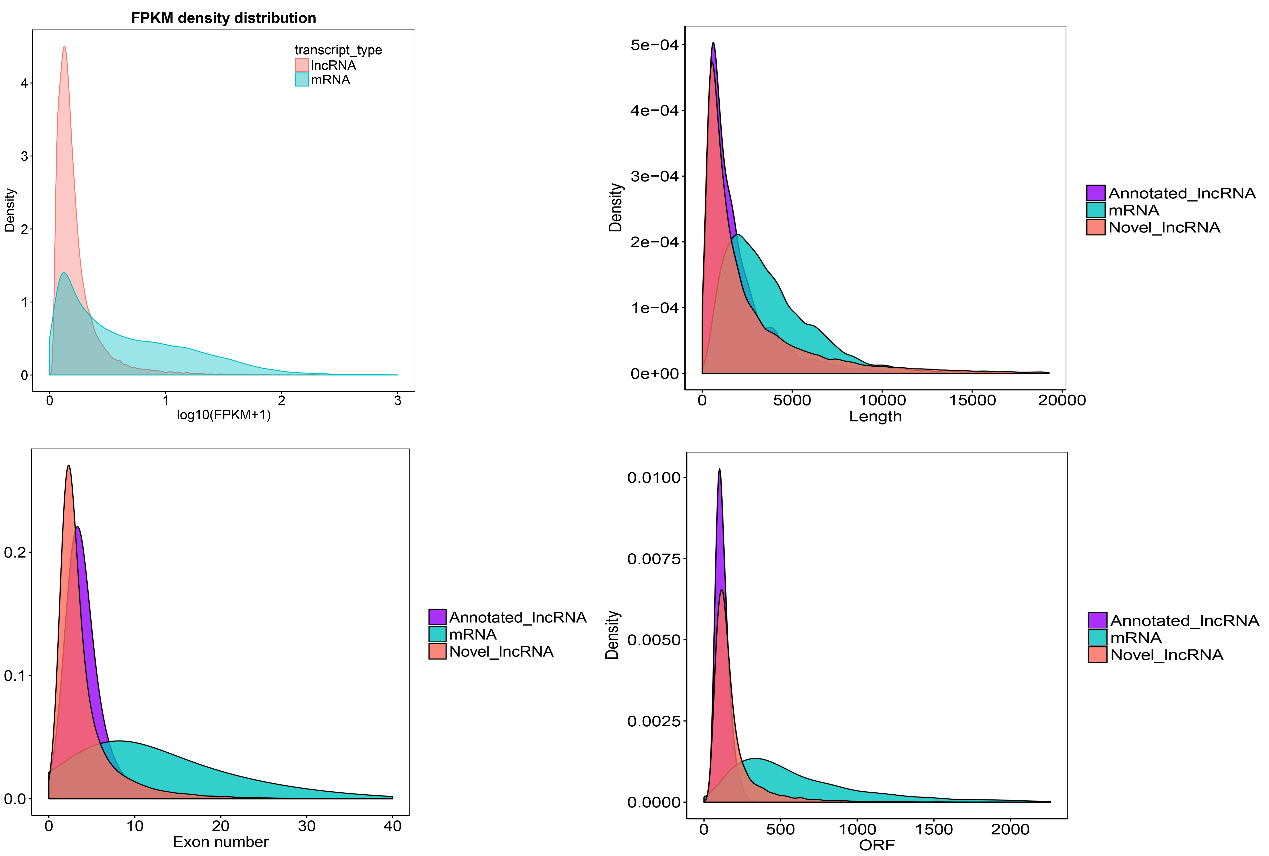


**Supplementary Figure 3.** Violin map of lncRNA compared with mRNA expression, length, number of exons and length of open reading frame

**Supplementary Table 1. Overview of sequencing data**

| **Sample name** | **Raw reads** | **Clean reads** | **Clean bases** | **Error rate**  **(%)** | **Q20**  **(%)** | **Q30**  **(%)** | **GC content (%)** |
| --- | --- | --- | --- | --- | --- | --- | --- |
| LG1 | 101807056 | 100801856 | 15.12G | 0.03 | 97.64 | 93.3 | 48.63 |
| LG2 | 98709390 | 96272778 | 14.44G | 0.03 | 97.65 | 93.39 | 49.39 |
| LG3 | 105458976 | 103497574 | 15.52G | 0.03 | 97.76 | 93.58 | 52.55 |
| WG1 | 113591524 | 112320412 | 16.85G | 0.03 | 97.93 | 93.88 | 49.69 |
| WG2 | 87555740 | 86085080 | 12.91G | 0.03 | 97.62 | 93.28 | 48.58 |
| WG3 | 93171146 | 91930790 | 13.79G | 0.03 | 97.84 | 93.68 | 48.47 |

Note: LG: Landes; WG: Sichuan White geese
