# Supplementary material for: Comparative Analysis of Testicular Histology and lncRNA–mRNA Expression Patterns Between Landes Geese (Anser anser) and Sichuan White Geese (Anser cygnoides)
Source: Front Genet. 2021 Mar 2;12:627384. doi: 10.3389/fgene.2021.627384 (PMC7963104; doi:10.3389/fgene.2021.627384)
Supplement: Supplementary file 7 [file Image_3.pdf]

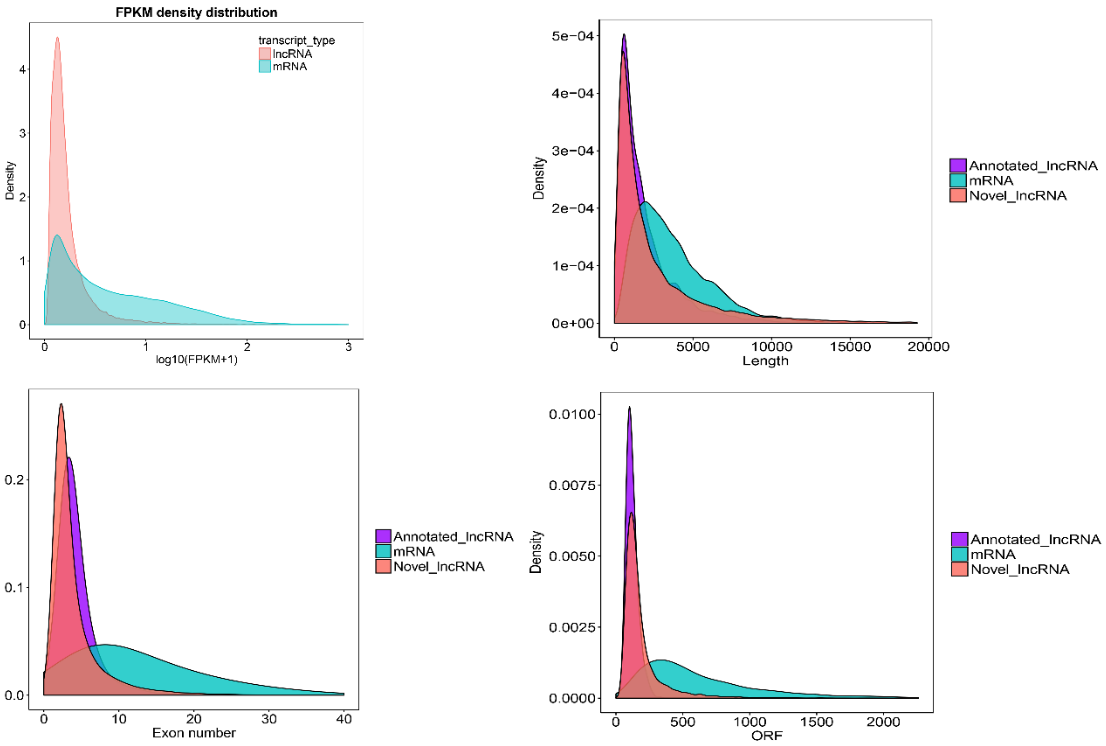

Figure S3. Violin map of lncRNA compared with mRNA expression, length, number of exons and length of open reading frame
